# Genome-wide cataloging and analysis of alternatively spliced genes in cereal crops

**DOI:** 10.1186/s12864-015-1914-5

**Published:** 2015-09-21

**Authors:** Xiang Jia Min, Brian Powell, Jonathan Braessler, John Meinken, Feng Yu, Gaurav Sablok

**Affiliations:** Department of Biological Sciences, Youngstown State University, Youngstown, OH 44555 USA; Center for Applied Chemical Biology, Youngstown State University, Youngstown, OH 44555 USA; Department of Computer Science and Information Systems, Youngstown State University, Youngstown, OH 44555 USA; Plant Functional Biology and Climate Change Cluster (C3), University of Technology Sydney, PO Box 123, Broadway, NSW 2007 Australia; Present address: Center for Health Informatics, University of Cincinnati, Cincinnati, OH 45267-0840 USA

**Keywords:** Alternative splicing, Cereal crops, Expressed sequence tags, mRNA

## Abstract

**Background:**

Protein functional diversity at the post-transcriptional level is regulated through spliceosome mediated pre-mRNA alternative splicing (AS) events and that has been widely demonstrated to be a key player in regulating the functional diversity in plants. Identification and analysis of AS genes in cereal crop plants are critical for crop improvement and understanding regulatory mechanisms.

**Results:**

We carried out the comparative analyses of the functional landscapes of the AS using the consensus assembly of expressed sequence tags and available mRNA sequences in four cereal plants. We identified a total of 8,734 in *Oryza sativa* subspecies (ssp) *japonica*, 2,657 in *O. sativa* ssp *indica*, 3,971 in *Sorghum bicolor*, and 10,687 in *Zea mays* AS genes. Among the identified AS events, intron retention remains to be the dominant type accounting for 23.5 % in *S. bicolor*, and up to 55.8 % in *O. sativa* ssp *indica*. We identified a total of 887 AS genes that were conserved among *Z. mays*, *S. bicolor*, and *O. sativa* ssp *japonica*; and 248 AS genes were found to be conserved among all four studied species or ssp. Furthermore, we identified 53 AS genes conserved with *Brachypodium distachyon*. Gene Ontology classification of AS genes revealed functional assignment of these genes in many biological processes with diverse molecular functions.

**Conclusions:**

AS is common in cereal plants. The AS genes identified in four cereal crops in this work provide the foundation for further studying the roles of AS in regulation of cereal plant growth and development. The data can be accessed at Plant Alternative Splicing Database (http://proteomics.ysu.edu/altsplice/).

**Electronic supplementary material:**

The online version of this article (doi:10.1186/s12864-015-1914-5) contains supplementary material, which is available to authorized users.

## Background

Spliceosome mediated post-transcriptional modifications are the biggest challenges in understanding and predicting the degree of certainty and complexity of the proteome diversity [1, 2]. One of the most important mechanisms that contribute to the diversity in the protein isoforms is alternative splicing (AS), thus modulating the protein function as a consequence of the linking of the functional units (exons and introns) in a ubiquitous manner [3]. In addition, to the observed alternative splicing sub-types such as exon skipping (ES), alternative donor (AltD) or acceptor (AltA) site, and intron retention (IR), various complex types can be formed by combination of basic events [4, 5]. Apart from the four basic events, alternative transcripts may arise as a consequence of the alternative transcription initiation, alternative transcription termination, and alternative polyadenylation [2]. AS isoforms might encode distinct functional proteins, or might be nonfunctional, which harbor a premature termination codon. These nonfunctional isoforms generated through the process called “regulated unproductive splicing and translation” are degraded by a process known as nonsense-mediated decay [6].

Previous reports estimated around 90 % of human genes containing multiple exons are alternatively spliced [7, 8]. In line with the observed reports in humans, alternative splicing has been shown to be a major player in generation of the plant proteome diversity with 60 % of *Arabidopsis thaliana* multi-exon genes undergoing alternative splicing [9]. Genome-wide identification and physiological implications of AS have been reported in a number of model and non-model plant species including *A. thaliana* [10–13], *Oryza sativa* [14], *Nelumbo nucifera* (sacred lotus) [15], *Vitis vinifera* [16], *Brachypodium distachyon* [5, 17]. AS transcripts are generally generated through three pathways: (1) IR in the mature mRNA; (2) alternative exon usage (AEU), resulting in ES; and (3) the use of cryptic splice sites that may elongate or shorten an exon that generates AltD or AltA site or both [14, 17]. Approximately 60–75 % of AS events occur within the protein coding regions of mRNAs, resulting changes in binding properties, intracellular localization, protein stability, enzymatic, and signaling activities [18]. In plants, IR has been shown to be the most dominant form with reports suggesting the proportions of intron containing genes undergoing AS in plants ranged from ~30 % to >60 % depending the depth of available transcriptome data [4, 5]. On contrast, recent reports suggest the down-regulation of the IR events and up-regulation of the alternative donor/acceptor site (AltDA) and ES under heat stress in model *Physcomitrella patens* [19]. With the advent of the Next Generation Sequencing (NGS) based approaches, fine scale physiological implications revealed alternative splicing as the prominent mechanism, which regulates the microRNA- mediated gene regulation by increasing the complexity of the alternative mRNA processing in *Arabidopsis* [20]. Complex networks of regulation of gene expression and variation in AS has played a major role in the adaptation of plants to their corresponding environment and additionally in coping with environmental stresses [13].

Rice (*O. sativa* ssp *japonica* and *indica)*, maize (*Zea mays*), and sorghum (*Sorghum bicolor*) are important cereal crops as major sources of food in many countries. Previously several approaches have widely demonstrated the identification of the quantitative trait loci, genes and proteins linked to the functional grain content in these species [21]. However, a major portion of the gene functional diversity is controlled by a spliceosomal regulated AS. AS has been shown to be a critical regulator in grass clade, demonstrating several of the genes involved in flowering and abiotic stress depicting alternative splicing [4, 17, 22]. Identifying alternative splicing genes in these cereal plants is the first step toward understanding the functions and regulations of these genes in plant development and abiotic or biotic stress resistance. Previously, using the homology based mapping approach and expressed sequence tags (ESTs) representing the functional transcripts, we identified a total of 941 AS genes in *B. distachyon,* a model temperate grass [5, 17]. Previous and recent reports on the identification and prevalence of the alternative splicing events in *O. sativa* [4, 23], *S. bicolor* [24], and *Z.* mays [25] have shown the functional diversity changes through EST/RNA-seq approaches. Previous report by Ner-Gaon et al. suggested a 3.7-fold difference in AS rates between *O. sativa* and *S. bicolor* using EST pairs gapped alignment [26]. The lack of the identification of the comparative AS events in cereal plants and realizing the importance of these functional foods in climate changes, we attempted to carry out the large scale analysis using the so far currently ESTs and mRNA based information in cereal plants to identify species specific and conserved AS events across cereal plants. In this work, we compared the AS event landscape and the AS gene functional diversity in cereal plants, which includes *O. sativa* ssp *japonica* and *indica*, *S. bicolor* and *Z. mays*, with a much deeper coverage of the identified AS events and also comparatively analyzed these AS genes with AS genes identified from *B. distachyon* to reveal conserved patterns of the AS across the grass species. Identified AS events will allow for the experimental characterization of the AS genes involved in important physiological processes. Investigation of the genome-wide conserved AS events across different species will shed light on the understanding of the evolution of the functional diversity in cereal plant for crop improvement.

## Methods

### Sequence datasets and sequence assembly

To identify the putative functional transcriptional changes across the Panicoideae lineage, we systematically queried and downloaded expressed sequence tags (ESTs) and mRNA sequences of *O. sativa* ssp *japonica* and *indica, S. bicolor*, and *Z. mays* from the dbEST and nucleotide repository of National Center for Biotechnology Information (NCBI; www.ncbi.nlm.nih.gov). Prior to aligning the ESTs/mRNAs to the corresponding genomic sequence, we applied stringent cleaning procedure using the strategy outlined below: 1) ESTs and mRNA sequences were subsequently cleaned using EMBOSS “trim” tool for trimming of the polyA or polyT ends; 2) Cleaned and trimmed ESTs and mRNA sequences were blasted using the BLASTN against UniVec and *E. coli* database for removal of vector and *E. coli* contaminants; 3) BLASTN searches against the plant repeat database which was built with TIGR gramineae repeat data and species specific repeat data including sorghum, maize, and rice available from ftp://ftp.plantbiology.msu.edu/pub/data/TIGR_Plant_Repeats/. Following stringent cleaning procedure, we assembled rice and sorghum cleaned EST and mRNA sequences using CAP3 with the following parameters: −p 95 –o 50 –g 3 –y 50 –t 1000 [27]. In case of the maize data, owing to the large number of available ESTs for this species, which is difficult to assemble, we followed an alternative way of assembling those ESTs. We first mapped ESTs and mRNA sequences to each individual chromosome of the maize genome using GMAP with default settings [28], and then chromosome specific-mapped ESTs and mRNAs were assembled individually using CAP3 with the parameters as mentioned above. The unmapped data and all assembled data from each individual assembly were combined and then re-assembled using CAP3 to generate a final consensus assembly for the further identification of the AS events. The raw data and assembled data for each organism were summarized in Table 1. For the prediction of the AS events, genome sequences, predicted protein coding DNA sequences (CDS), and related GFF data of *O. sativa* ssp *japonica*, *Z. mays*, and *S. bicolor* were downloaded from Phytozome database (http://www.phytozome.net/) [29–32]. The genome sequences and CDS data of *O. sativa* ssp *indica* (strain 93–11) were downloaded from BGI database (http://rise2.genomics.org.cn/page/rice/index.jsp) [33].

**Table 1:** Summary of raw sequence data and assembled data in each organism

Species	ESTs	mRNAs	Total Sequences	Cleaned Sequences	Total PUTs	Average Length (bp)
*O. sativa ssp japonica*	987327	82451	1069868	1053842	163778	783
*O. sativa ssp indica*	207012	11953	219065	212768	102424	751
*S. bicolor*	209835	33248	243083	241690	60189	1002
*Z. mays*	2019524	91990	2111514	1822653	488243	466

*PUTs* putative unique transcripts

### Putative unique transcripts to genome mapping, identification and functional annotation of AS isoforms

In the present study, taking into the account the genome duplication events in *Z. mays* and *S. bicolor*, accurate prediction of the alternative splicing events is a major concern over the decades. In our study, calling and predicting alternative splicing events is taken into account by mapping of EST and mRNA assemblies, i.e. putative unique transcripts (hereafter simply referred them as PUTs), to the corresponding genomic sequences were carried out using in-house developed algorithm, ASFinder (http://proteomics.ysu.edu/tools/ASFinder.html/) [34], which uses SIM4 program [35] to map PUTs to the corresponding genome and then subsequently identifies those PUTs that are mapped to the same genomic location but have variable exon-intron boundaries as AS isoforms. To avoid the call of the spurious alternative splicing events, we applied a threshold of minimum of 95 % identity of aligned PUT with a genomic sequence, a minimum of 80 bp aligned length, and >75 % of a PUT sequence aligned to the genome [17]. Application of the above identity percentage and the aligned length removes the chance of the false positive AS events calling as a result of genome duplication events. The output file (AS.gtf) of ASFinder was then subsequently submitted to AStalavista server (http://genome.crg.es/astalavista/) for AS event analysis [36]. The percentage of alternative splicing genes was estimated using the genome predicted gene models having alternative splicing PUT isoforms among total genes models having at least one PUT, the results were presented in Table 2.

**Table 2:** Percentage of alternative splicing genes

	Total mapped PUTs (%)	PUT match to gene model	Total unique genes	AS genes	AS (%)
*O. sativa ssp japonica*	104447 (63.8)	71830	26191	7883	30.1
*O. sativa ssp indica*	47843 (46.7)	36467	17402	2414	13.9
*S. bicolor*	50224 (83.4)	38654	26540	3580	13.5
*Z. mays*	207332 (42.5)	119418	28698	9689	33.8

*AS* Alternative splicing

We further queried the coding potential and corresponding coding frame of each PUT using the ORFPredictor [37], and to assess the full–length transcript coverage using TargetIdentifier [38] as previously described. Functional classification was assigned to the PUTs by performing BLASTX searches with an E-value threshold of 1E-5 against UniProtKB/Swiss-Prot. Predicted protein sequences from ORFPredictor were further annotated using rpsBLAST against the PFAM database (http://pfam.xfam.org/). Gene Ontologies (GOs) were assigned on the basis of the functional homology obtained by the BLASTX searching algorithm against the UniProtKB/Swiss-Prot. The GO categories were further analyzed using GO SlimViewer using plant specific GO terms [39]. To assess the functional coverage of the assembled PUTs, we further compared PUTs against the predicted gene primary transcripts using BLASTN with a cut off E-value of 1E-10, ≥ 95 % identity and minimum aligned length of 80 bp.

### Conserved alternatively spliced genes in cereal plants and visualization of AS

For the identification of the potentially conserved AS genes among *O. sativa* ssp *japonica* and *indica*, *Z. mays* and *S. bicolor*, reciprocal BLASTP (cutoff E-value 1E-10) were done using the longest (or longer) ORF of the AS PUT isoforms for classifying the conserved AS pairs between species or sub-species. Venn graphical visualization for conserved AS pairs were obtained using R programming language (http://www.r-project.org/). Visualization of the alternative splicing events with genome tracks is critically important from two points of views: (1) To have a graphic look at the corresponding genomic coordinate and associated genic functional changes; and (2) To extract the corresponding spliced region of interest for functional primer designing of putative AS events. Keeping in view the above points, AS events identified in this study along with the integrated genomic tracks are available from Plant Alternative Splicing Database (http://proteomics.ysu.edu/altsplice/) [15, 17]. The specific pages associated with the cereal plants offer several end-users functionalities such as querying using the PUT ID, gene ID, keywords in functional annotation, PFAM, or AS event types as “*query fields*”. Additionally, the identified AS events can be visualized and compared with predicted gene models using GBrowse for comparative assessment. Nevertheless, we also deployed BLASTN functionality to search for the PUTs and AS isoforms. The data analyzed along with the GO and PFAM annotations in the present research are publicly available at: http://proteomics.ysu.edu/publication/data/.

## Results and discussion

### EST assembly and annotation

Optimization of the assembly parameters and mapping functionally annotated PUTs is a key parameter to provide a robust identification and classification of the AS events. Table 1 represents the assembly information, including the final cleaned reads for the assembly, mRNA count for each species, assembled consensus sequence and average length of assembled consensus. In the present research, we assembled and generated consensus PUTs accounting for a total of 163,778 PUTs in *O. sativa* ssp *japonica*, 102,424 PUTs in *O. sativa* ssp *indica*, 60,189 PUTs in *S. bicolor*, and 488,243 PUTs in *Z. mays*. The average length (N50) of assembled PUTs was 783 bp in *O. sativa* ssp *japonica*, 751 bp in *O. sativa* ssp *indica*, 1,002 bp in *S. bicolor*, and 466 bp in *Z. mays*. To check for the coverage of the assembled functional transcriptome, we further checked for the functional assignments and all the assembled PUTs were structurally and functionally annotated including putative open reading frame (ORF) prediction, coding region full-length prediction, a putative function and PFAM prediction, which ensures the reliability of the assembly strategies in case of large complex ploidy genomes underwent whole genome duplication events. PUTs were mapped to their corresponding genomes and predicted gene models were also visualized using GBrowse.

### Gapped alignments of PUTs to genome, detection and classification of alternative splicing events

Following the sequence assembly, resulting unique PUTs were mapped onto their corresponding genomic sequences using gapped alignments as implemented in SIM4 method that was integrated as part of ASFinder [34]. The numbers of mapped PUTs and matched gene models, as well as the number of the observed AS genes are presented in Table 2. We observed that a relatively larger proportion of PUTs in *S. bicolor* (83.4 %) and *O. sativa* ssp *japonica* (63.8 %) aligned to their genomes as compared to the other cereal plants. We identified a total of 8,734 in Oryza sativa subspecies (ssp) japonica, 2,657 in O. sativa ssp indica, 3,971 in Sorghum bicolor, and 10,687 in Zea mays AS genes (Table 3). The percentage of AS genes was estimated based on the proportion of predicted gene models having AS PUT isoforms over the total gene models having an EST (PUT) evidence (Table 2). The percentages of AS genes vary in different cereal plants, up to 30.1 % in *O. sativa* ssp *japonica* and 33.8 % in *Z. mays*, and relatively low in *O. sativa* ssp *indica* (13.9 %) and in *S. bicolor* (13.5 %). The difference in the mapping rate and AS rate might be due to the difference in the number of ESTs available for respective species. Previous reports on AS in *B. distachyon* clearly illustrates the fact that availability of the more ESTs/mRNAs reflects the prediction of the AS landscape [5, 17].

**Table 3:** Alternative splicing events in different cereal species

Species	IR(%)	AltD(%)	AltA (%)	ES (%)	Complex event (%)	Total events	Total AS genes
*O. sativa ssp japonica*	8288 (42.0)	1245 (6.3)	1950 (9.9)	762 (3.9)	7447 (37.8)	19692	8734
*O. sativa ssp indica*	2193 (55.8)	332 (8.5)	576 (14.7)	161 (4.1)	665 (16.9)	3927	2657
*S. bicolor*	4448 (23.5)	1072 (5.7)	1230 (6.5)	507 (2.6)	11681 (61.7)	18938	3971
*Z. mays*	11048 (40.4)	2080 (7.6)	3314 (11.4)	1568 (5.7)	5576 (20.4)	23386	10687

*IR* Intron Retention, *AltD* Alternative donor, *AltA* Alternative acceptor, *ES* exon skipping

Recent reports using the RNA-seq technology revealed that AS is common in plants—around 61 % of multi-exonic genes in *A. thaliana* are alternatively spliced under normal growth conditions [12], and ~40 % of intron containing genes that undergo AS in maize [25]. Classification of the AS events observed in the cereal plants are listed in Table 3 showing the prevalence of the IR as the major splicing type showing frequency as high as 55.8 % in *O. sativa* ssp *indica* and as low as 23.5 % in *S. bicolor* (Table 3). The high frequency of the IR in the mature mRNA is perfectly in line with the previously observed frequencies of IR (30–50 %) in AS landscape in *A. thaliana* and *O. sativa* [14]. It is worthwhile to mention that plant spliceosomal machinery supports the intron definition model, thus identifies the introns for pre-mRNA splicing as oppose to the abundant exon-spliceosome model observed in case of mammals. Previous arguments have clearly justified the cause and benefits of retaining the introns as potential cytoplasmic translatable transcripts [26] or as mediators of increasing the gene expression, a process widely described as intron-mediated enhancement (IME) of gene expression [40]. The abundance of IR as a major AS event is consistent with previous reports including *Medicago tuncatula* (39 %), *Populus trichocarpa* (34 %), *A. thaliana* (56 %), *O. sativa* (54 %), *Chlamydomonas reinhardtii* (50 %), *Z. mays* (58–62 %) and *B. distachyon* (55.5 %) [14, 17, 25, 41, 42]. In contrast, recently IR has been found remarkably repressed under elevated temperature in *P. patens* [19].

Alternative acceptor (AltA) and donor (AltD) represent the second most abundant and classified functional class of observed AS events with AltA showing a relatively higher frequency as compared to AltD (Table 3). Although ES events have been described as the rarest events in plants, which are in line with the observed results in this study, recently they have been proposed as the candidates of the transgene regulation using the conditional splicing [43]. We noted that 61.7 % events are complex events in sorghum, which have more than one basic event in compared paired PUTs. This is clearly related to the relative longer lengths of the PUTs in sorghum assembly. Recent reports suggest the differential up-regulation of the alternative donor/acceptor site (AltDA) and ES elucidating the importance of these events as indicators of early heat stress [19].

Our data in this work clearly showed that the number of AS genes and the percentage of genes with AS are different in different crops (Tables 2 and 3). However, this observation only reflects the current state in these plants based on the available data. Our previous analysis on AS in *B. distachyon* clearly demonstrated that more AS genes were identified with more available ESTs/mRNA data [5, 17]. This is also consistent with the finding of increasing frequency of occurrence of AS in Arabidopsis with time—a reflection of an accumulation of available transcriptome data, for example, only 1.2 % of the genes in Arabidopsis were reported undergo AS in 2003 and now it was estimated over 60 % of intron-containing genes undergo AS [13].

### Features of exons and introns in protein coding genes: indicators of gene evolution

**Fig. 1:**
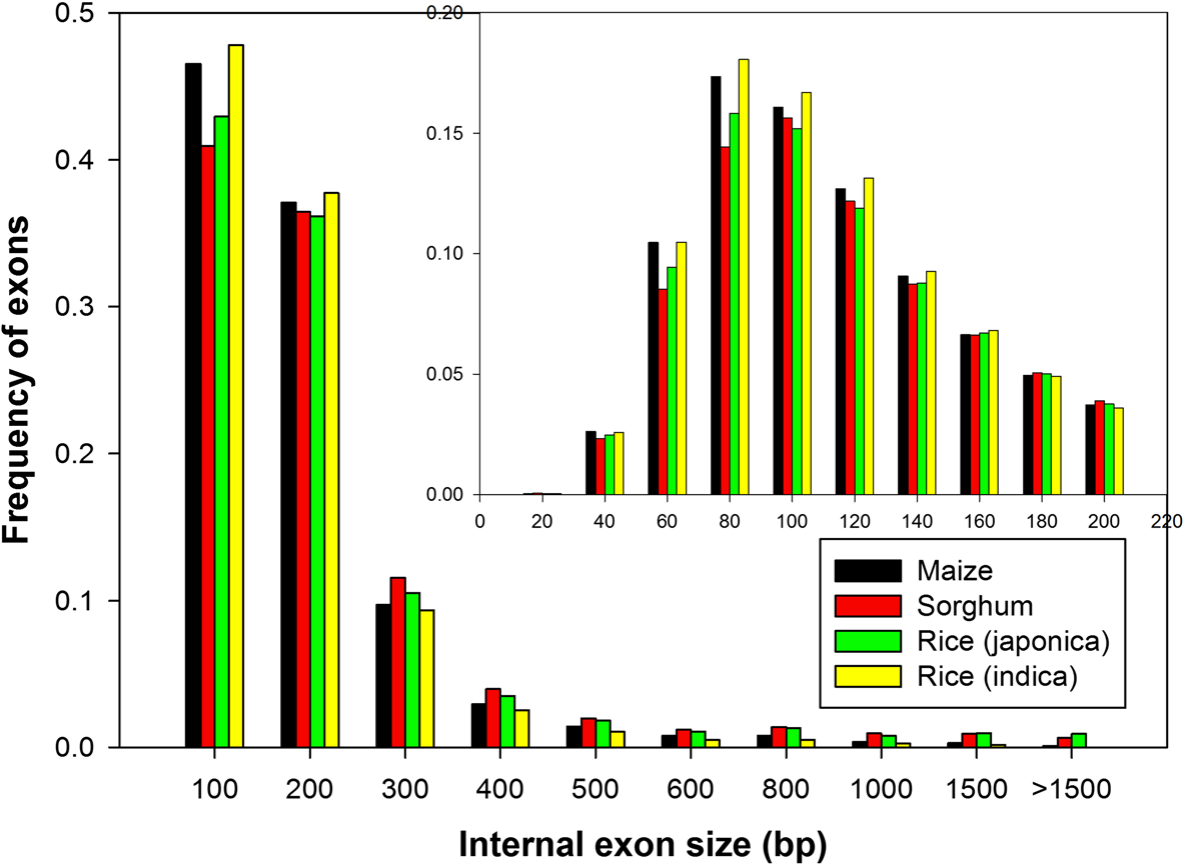
Distribution of internal exon size: The x-axis indicates the size of internal exons. Bin sizes are right inclusive (e.g., bin 100 comprises sequences of lengths 1–100 bp). The y-axis indicates the frequency of internal exons. The inset shows a detailed distribution of small internal exons

**Fig. 2:**
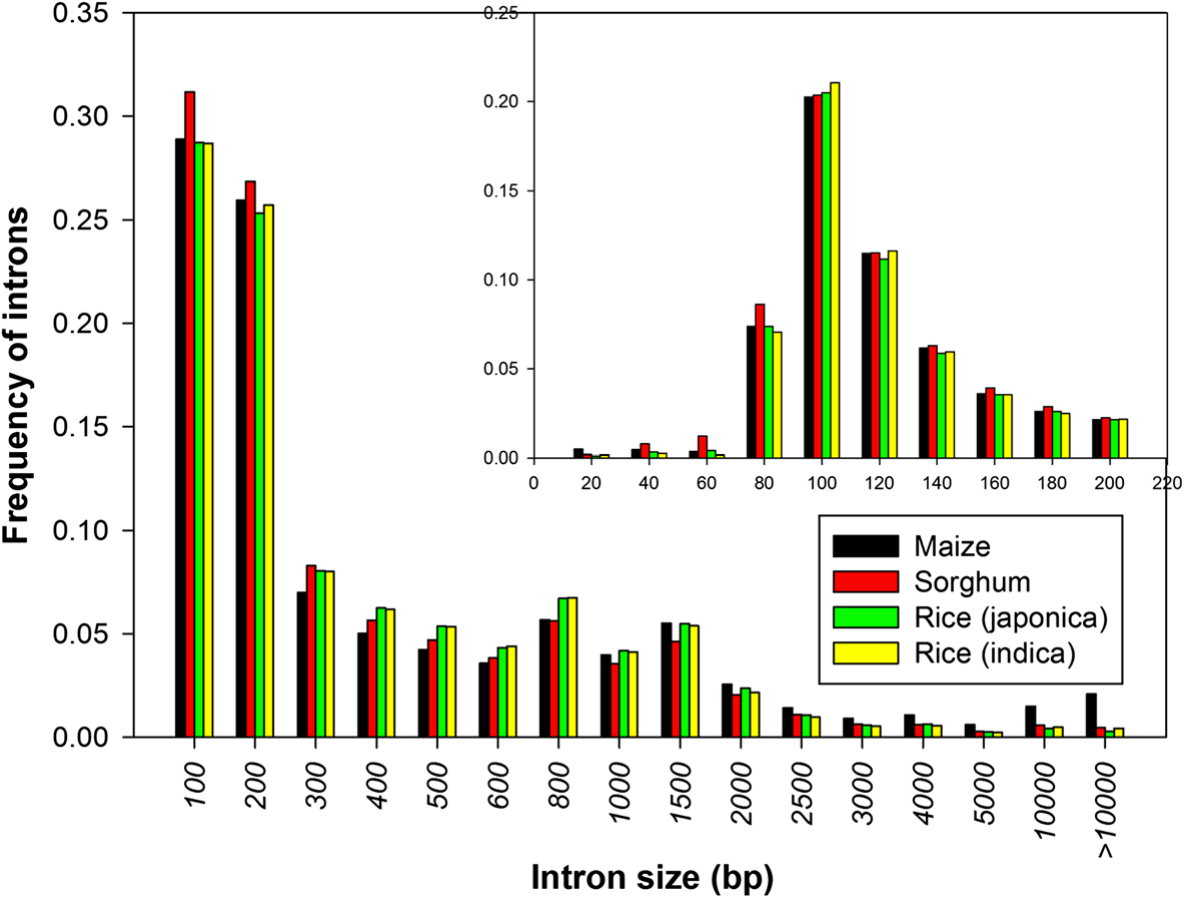
Distribution of intron size: The x-axis indicates the size of introns. Bin sizes are right inclusive (e.g., bin 100 comprises sequences of lengths 1 –100 bp). The y-axis indicates the frequency of introns. The inset shows a detailed distribution of small introns

Understanding the patterns of gene evolution and identifying signatures of convergent and divergent evolution is of paramount importance, especially when we are addressing the genome complexity in terms of gene evolution. Exon-intron framework properties such as length distribution and GC content evolution have been previously used to demonstrate the gene evolution [44]. Additionally, longer introns as compared to short introns have been shown to play an important role in the gene expression [40, 45]. However, reports by Yang [46] demonstrate the negative correlation of the long introns with the levels of the expression in *A. thaliana* and *O. sativa*. Realizing the importance of the features of exon-intron in evolution and physiological responses, we extracted and plotted the length distribution of all internal exons and introns from each plant and the results are summarized (Table 4; Fig. 1; Fig. 2). Interestingly, we observed that the average internal exon lengths in *O. sativa* ssp *indica* and *Z. mays* are almost similar, and are relatively much shorter than the internal exon lengths in *O. sativa* ssp *japonica* and *S. bicolor*. On the other hand, *Z. mays* had the longer intron length (554 bp) and showed a wide variation in intron lengths as compared to the observed range of intron lengths (422–440 bp) in other three cereal plants in this study. We further analyzed deeply the exon size and intron size distribution frequencies demonstrating that *Z. mays* and *O. sativa* ssp *indica* had a relatively much higher proportion of internal exons of a smaller size (<120 bp) (Fig. 1). The observed frequency of internal exon lengths below 300 bp was 0.93 in *Z. mays*, 0.95 in *O. sativa* ssp *indica*, 0.89 in *S. bicolor*, and 0.90 in *O. sativa* ssp *japonica. S. bicolor* and *O. sativa* ssp *japonica* displayed more exons of relatively large size, whereas *Z. mays* displayed a higher number of long introns (Fig. 2). Prevalence of the introns richness and specifically long introns have been previously been shown to be widely associated with the increased expression of *Adh*1, *Sh*1, *Bz*1, *Hsp82*, actin, and *GapA*1 genes in *Z.* mays [47–51] and *salT*, *Act*1, and *tpi* genes in rice [52, 53]. Additionally, a relative higher proportion of introns having a shorter length were observed in *S. bicolor*. We also observed ~2 % introns in maize and a small number of introns (<0.5 %) in other plants having a size >10 kb. However, taking into account the possible errors in PUT and genome assembly, these long introns were not included in the calculation of the average intron size. It is worthwhile to mention that the average internal exon size (180 bp) and intron size (440 bp) in *O. sativa* ssp *japonica* obtained in this work were close to the exon (193 bp) and intron (433 bp) size obtained previously in *O. sativa*, which presents the robustness of the implemented approach [14].

**Table 4:** Exon and intron size in cereal plants

	Exon			Intron		
	Sample size	Average size (bp)	SD	Sample Size	Average size (bp)	SD
*O. sativa ssp japonica*	127627	180	261	180575	440	695
*O. sativa ssp indica*	52330	133	113	79735	434	703
*S. bicolor*	106753	179	222	144860	422	747
*Z. mays*	137020	142	133	209139	554	1057

*SD* Standard deviation

### Functional classification of AS genes

AS and gene regulation can be observed at almost all levels of biological interactions [54]. The AS transcripts identified in the present study were functionally annotated for the Gene Ontologies (GOs) and for putative protein domains association by performing a BLASTX search of all PUTs against UniProt/Swiss-Prot database. The ORFs of PUTs were identified using ORFPredictor webserver [37]. The protein families of the AS genes, using the longest ORF of each AS gene, were predicted using rpsBLAST searching PFAM database. Among predicted ORFs of these AS genes, 6,900 in *Z. mays*, 4,939 in *O. sativa* ssp *japonica*, 1,362 in *O. sativa* ssp *indica*, and 2,890 in *S. bicolor* were classified with a putative protein family (Table 5, Additional file 1: Table S1). We further classified AS gene functional products into 2,030 unique protein families in *Z. mays*, 1,708 unique protein families in *O. sativa* ssp *japonica*, 757 unique protein families in *O. sativa* ssp *indica*, and 1,194 unique protein families in *S. bicolor*. Among the protein functions, encoded by these AS genes, widely includes protein kinase domain, RNA recognition motif, protein tyrosine kinase, ring finger domain, cytochrome P450, Myb-like DNA-binding domain, WRKY DNA-binding domain, Thioredoxin and protein phosphatase 2C (Table 5). A complete list of all the protein families encoded by AS genes is shown in Additional file 1: Table S1. Our analysis demonstrated that AS genes in cereal plants encode diverse protein families that play important roles in various biological processes. A classical example can be WRKY- DNA binding domains, which represents the largest and functionally diverse transcription factors in plants playing a major role in developmental and physiological processes. Previous studies have widely demonstrated the presence of the alternative ORF in the WRKY genes [55, 56]. Yang et al. [57] and Feng et al. [58] have clearly highlighted the role of the alternative splicing and WRKY in plant immunity. Previous functional studies have shown the presence of the splicing of the R-type intron and V-type intron in *O. sativa* WRKY genes and functionally correlated them to plant immunity [59]. MYB-domains play an important role in plant defense mechanism and are transcriptionally regulated by alterative splicing in *A. thaliana* and *O. sativa* and encode MYB- or MYB-related proteins [60]. Alternative splicing of MYB related genes *MYR*1 and *MYR2* have clearly demonstrated the change in protein dimerization and folding as a consequence of alternative splicing thus affecting the transcriptional sensitivity in light mediated responses [61].

**Table 5:** Protein family classification of alternative genes in cereal plants

PFAM	Domain	*Z. mays*	*O. sativa ssp japnoica*	*O. sativa ssp indica*	*S. bicolor*	Putative Functions
pfam00069	Pkinase	205	228	55	74	Protein kinase domain
pfam00076	RRM_1	112	61	32	43	RNA recognition motif
pfam07714	Pkinase_Tyr	88	79	12	25	Protein tyrosine kinase
pfam13639	zf-RING_2	53	28	8	15	Ring finger domain
pfam00067	p450	45	56	4	37	Cytochrome P450
pfam00481	PP2C	45	25	11	11	Protein phosphatase 2C
pfam00249	Myb_DNA-binding	44	14	7	22	Myb-like DNA-binding domain
pfam00179	UQ_con	43	17	10	7	Ubiquitin-conjugating enzyme
pfam00010	HLH	41	5	1	7	Helix-loop-helix DNA-binding domain
pfam00071	Ras	38	20	11	7	Ras family
pfam00141	peroxidase	37	30	11	31	Peroxidase
pfam00153	Mito_carr	35	24	7	14	Mitochondrial carrier protein
pfam01559	Zein	35	0	0	0	Zein seed storage protein
pfam01490	Aa_trans	33	12	1	7	Transmembrane amino acid transporter protein
pfam02365	NAM	33	33	8	14	No apical meristem (NAM) protein
pfam00125	Histone	31	9	3	5	Core histone H2A/H2B/H3/H4
pfam01370	Epimerase	31	26	8	22	NAD dependent epimerase/dehydratase family
pfam00083	Sugar_tr	30	22	7	10	Sugar (and other) transporter
pfam00847	AP2	30	9	3	9	AP2 domain
pfam00106	adh_short	29	25	10	15	short chain dehydrogenase
pfam00657	Lipase_GDSL	29	5	1	16	GDSL-like Lipase/Acylhydrolase
pfam00085	Thioredoxin	28	14	6	11	Thioredoxin
pfam00226	DnaJ	28	18	9	9	DnaJ domain
pfam03151	TPT	27	9	2	6	Triose-phosphate Transporter family
pfam00004	AAA	26	18	6	14	ATPase family associated with various cellular
pfam00270	DEAD	24	21	5	8	DEAD/DEAH box helicase
pfam00504	Chloroa_b-bind	24	19	11	20	Chlorophyll A-B binding protein
pfam02309	AUX_IAA	24	13	5	5	AUX/IAA family
pfam00149	Metallophos	23	9	3	13	Calcineurin-like phosphoesterase
pfam00134	Cyclin_N	22	9	2	4	Cyclin
pfam00450	Peptidase_S10	21	18	6	18	Serine carboxypeptidase
pfam03106	WRKY	21	22	7	7	WRKY DNA -binding domain
pfam13041	PPR_2	21	30	1	12	PPR repeat family
Total		6900	4939	1362	2890	

Note: a complete list is shown in Additional file 1: Table S1

GO analysis according to biological and molecular function revealed a wide visibility in all the major biological and molecular functions (Table 6; Table 7). Interestingly, even the data we collected are from pooled data in the public domain, i.e., not from a strictly controlled experiment, our GO analysis revealed that relative to the average of AS percentage, a higher percentage of genes involved in response to abiotic stimulus, photosynthesis, carbohydrate metabolic process, and cell death are involved in AS in cereal plants. In contrast, the genes involved in multicellular organismal development and reproduction had a lower percentage of AS (Table 6). GO molecular function analysis revealed that genes encoding proteins having DNA binding, sequence-specific DNA binding transcription factor activity, nuclease activity had a lower percentage of AS, and the gene coding proteins for protein binding and having kinase activity had a higher percentage of AS in the majority of plants (Table 7). Our observed results are consistent with literature reviewed recently by Reddy et al. [4] and Staiger and Brown [22] that AS is involved in most plant processes and plays regulated roles in plant development and stress responses.

**Table 6:**
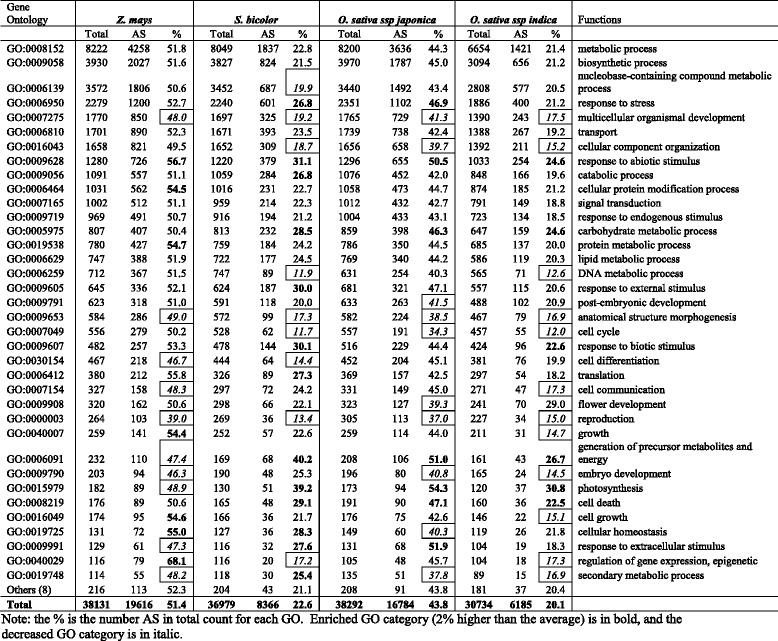
Classification of biological processes based on Gene Ontology (GO)

**Table 7:**
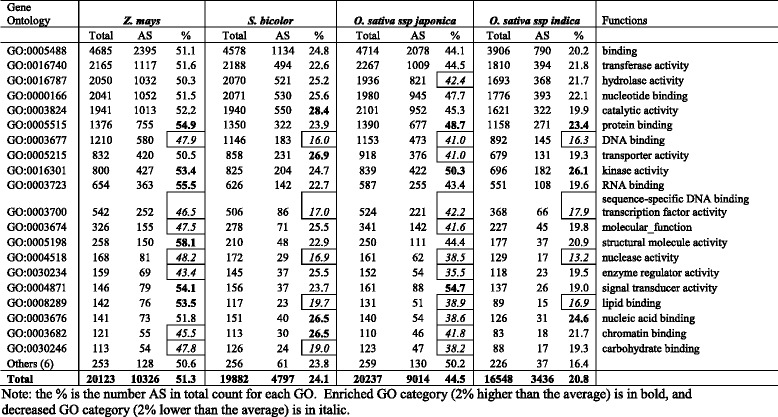
Classification of molecular functions based on Gene Ontology (GO)

### Conserved alternatively spliced genes

**Fig. 3:**
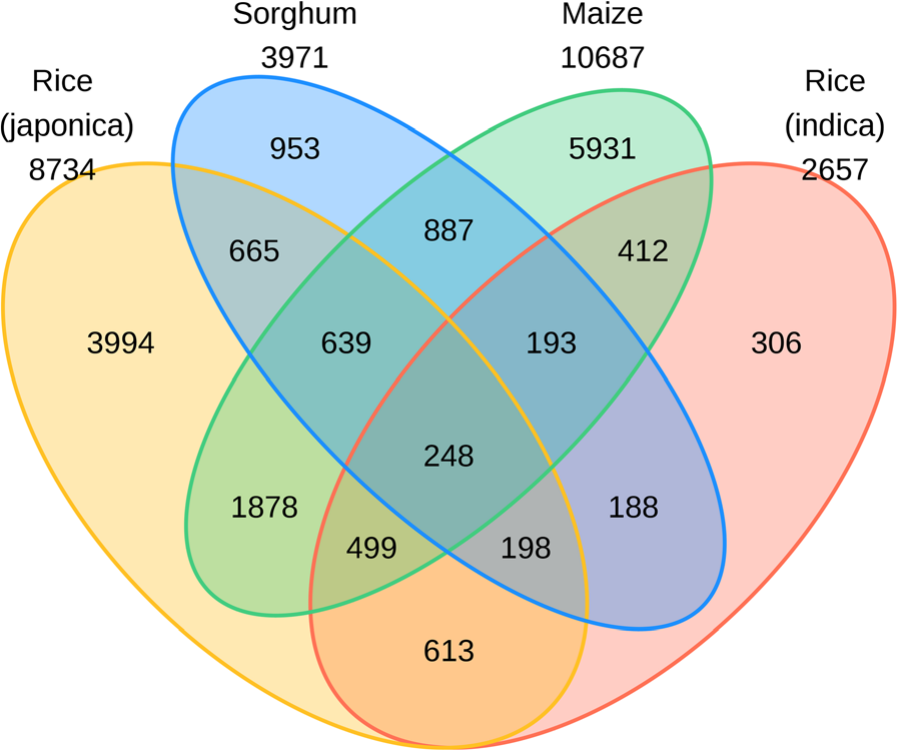
Conserved alternative splicing genes in rice (*Oryza sativa*) ssp *japonica*, rice ssp *indica*, sorghum (*Sorghum bicolor*), and maize (*Zea mays*) plants

Classification of the conserved alternative splicing events provides a framework for understanding the evolution of the functional genes and their genic-regulation at the transcriptional level, which may initiate the cross-talks between the evolution of the genes under AS and between the transcriptional environment and the ecological adaptation. For the identification of the conserved AS pairs, longest ORFs of AS genes in each studied species were compared using the BLASTP (cutoff E-value 1E-10) to identify the best-reciprocal top hit as the conserved pairs. In total, we identified 1558 AS genes conserved between *O. sativa* ssp *japonica* and *indica*, 3,246 AS genes conserved between *O. sativa* ssp *japonica* and *Z. mays*, and 1,967 AS genes between *S. bicolor* and *Z. mays* (Additional file 2: Table S3). A total of 887 AS genes are conserved among *Z. mays*, *S. bicolor*, and *O. sativa* ssp *japonica*. More importantly, we identified 248 AS genes conserved among all four plants (Fig. 3). Furthermore, using the same approach, we identified a total of 53 AS genes conserved with *B. distachyon* belonging to BEP-clade of grass evolution. The co-orthologous conserved 53 AS genes are listed in Table 8. The set of co-orthologs 248 AS genes conserved in the four plants, with 53 of them conserved to *B. distachyon,* are provided in Additional file 3: Table S2 (can be downloaded at http://proteomics.ysu.edu/altsplice/). Interestingly, one of the candidates among the conserved gene is Drought-induced protein (*Di*19). It has been previously suggested that the presence of the retained intron within the coding sequence may give rise to the non-sense mediated decay (NMD) [62]. Recent studies highlight the role of cycloheximide in introducing pre-mature termination codons (PTCs) and NMD in *A. thaliana Di*19, indicating the splicing mechanism in Di19 [63]. Identification of the *Di*19 mediated splicing will be of critical importance in increasing the drought resistance or increasing the captive yield of the cereal plants, which are acting as major suppliers of food in climate change. As current analysis were based on the pooled EST/mRNA sequences available in the public domain, more biologically functionally conserved AS genes will be identified when more transcriptome data are collected with improved technologies, various environmental conditions, developmental stages and tissues in these cereal crops. The present data is of immense potential for experimental validation and highlights the role of the AS and biological significance in plant, growth development and environmental regulation, which is a standing challenge in climate change.

**Table 8:** Conserved alternative splicing genes among five monocot plants

*O. sativa ssp indica*	*O. sativa ssp japonica*	Z. mays	S. bicolor	*B. distachyon*	CDD/Pfam		
Osi19962	Osj954	Zm92934	Sb6267	Bd2565	pfam03171	2OG-FeII_Oxy	2OG-Fe(II) oxygenase superfamily
Osi18787	Osj44013	Zm40020	Sb12294	Bd28385	pfam00004	AAA	ATPase family associated with various cellular
Osi6875	Osj22392	Zm88316	45969421	Bd7352	pfam00248	Aldo_ket_red	Aldo/keto reductase family
Osi9356	Osj41340	Zm162	Sb17314	Bd6214	pfam00248	Aldo_ket_red	Aldo/keto reductase family
CX100091	Osj15328	Zm35072	Sb10885	Bd29210	pfam00439	Bromodomain	Bromodomain
Osi12568	Osj24409	Zm100060	Sb8817	Bd24009	pfam05042	Caleosin	Caleosin related protein
CT843009	Osj14649	Zm32705	Sb6709	Bd10918	pfam00571	CBS	CBS domain
Osi524	CT828785.1	Zm73067	Sb4586	Bd10523	pfam04733	Coatomer_E	Coatomer epsilon subunit
Osi21096	Osj16673	FL103380	2.42E + 08	Bd4031	pfam07876	Dabb	Stress responsive A/B Barrel Domain
Osi8549	Osj47391	Zm69871	Sb334	Bd7166	pfam05605	Di19	Drought induced 19 protein (Di19)
CT833644.1	CI258157	Zm20082	Sb10226	Bd7036	pfam05057	DUF676	Putative serine esterase (DUF676)
Osi21136	Osj16693	Zm46142	Sb13903	Bd7810	pfam05623	DUF789	Protein of unknown function (DUF789)
Osi19974	Osj14932	Zm70017	Sb10575	Bd3731595	pfam00676	E1_dh	Dehydrogenase E1 component
Osi1759	Osj22934	Zm35625	Sb15873	Bd7027	pfam01370	Epimerase	NAD dependent epimerase/dehydratase family
CT842225	Osj27697	Zm91971	Sb4930	Bd268	pfam00316	FBPase	Fructose-1-6-bisphosphatase
Osi20900	Osj16392	Zm58947	Sb3303	Bd7531597	pfam00210	Ferritin	Ferritin-like domain
Osi339	Osj20205	Zm20714	Sb12056	Bd6374	pfam00762	Ferrochelatase	Ferrochelatase
Osi11082	Osj491	Zm59942	Sb3313	Bd27405	pfam00125	Histone	Core histone H2A/H2B/H3/H4
Osi13655	Osj36042	Zm81325	Sb15256	Bd28446	pfam00403	HMA	Heavy-metal-associated domain
Osi11360	Osj36865	Zm38497	Sb20674	Bd9583	pfam00447	HSF_DNA-bind	HSF-type DNA-binding
Osi17520	Osj35947	Zm27347	Sb9471	Bd7833	pfam01156	IU_nuc_hydro	Inosine-uridine preferring nucleoside
Osi13902	Osj25885	Zm23750	Sb12436	Bd28318	pfam00013	KH_1	KH domain
Osi11280	Osj28328	Zm35841	Sb9907	Bd13744	cd00116	LRR_RI	Leucine-rich repeats (LRRs)
CT844279	CB642464	Zm3338	Sb7337	Bd28467	pfam01717	Meth_synt_2	Cobalamin-independent synthase
Osi1437	Osj37916	Zm4695	Sb5119	Bd7994	pfam00635	Motile_Sperm	MSP (Major sperm protein) domain
Osi231	Osj25397	Zm37411	Sb10332	Bd28960	pfam14360	PAP2_C	PAP2 superfamily C-terminal
Osi8815	Osj32580	Zm61468	Sb11226	Bd6619	pfam01195	Pept_tRNA_hydro	Peptidyl-tRNA hydrolase
Osi16666	Osj19199	Zm104454	Sb12015	Bd16056	pfam00450	Peptidase_S10	Serine carboxypeptidase
Osi12736	Osj14309	Zm22618	Sb7927	Bd8683	pfam00141	peroxidase	Peroxidase
Osi833	Osj39350	Zm92939	Sb19533	Bd5931597	pfam00069	Pkinase	Protein kinase domain
Osi3301	Osj17780	Zm29726	Sb14730	Bd29285	pfam00069	Pkinase	Protein kinase domain
Osi6061	Osj15126	Zm59883	Sb673	Bd15932	PLN02756	PLN02756	S-methyl-5-thioribose kinase
Osi6187	Osj42201	Zm39790	Sb2138	Bd8363	pfam00348	polyprenyl_synt	Polyprenyl synthetase
Osi13092	Osj21144	Zm33939	Sb5787	Bd23758	pfam14299	PP2	Phloem protein 2
Osi11891	NM_001070568.2	Zm87952	Sb2001	Bd2595	pfam00854	PTR2	POT family
Osi20788	Osj19691	Zm39384	30944654	Bd10083	pfam07992	Pyr_redox_2	Pyridine nucleotide-disulphide
CT837906.1	Osj7689	Zm101865	Sb5520	Bd25885	pfam00719	Pyrophosphatase	Inorganic pyrophosphatase
Osi21504	Osj17274	Zm6058	Sb11340	Bd21664	pfam00072	Response_reg	Response regulator receiver domain
Osi15366	Osj24220	Zm5068	Sb10671	Bd23705	pfam02453	Reticulon	Reticulon
Osi9029	Osj47510	Zm24118	Sb11323	Bd8231593	pfam03214	RGP	Reversibly glycosylated polypeptide
Osi5643	Osj25267	Zm80771	Sb227	Bd11010	pfam01246	Ribosomal_L24e	Ribosomal protein L24e
Osi8310	Osj36859	Zm101179	Sb11303	Bd6311	pfam00076	RRM_1	RNA recognition motif
Osi1456	Osj43479	Zm371	Sb12579	Bd15819	pfam00076	RRM_1	RNA recognition motif
Osi773	Osj43052	Zm24001	Sb2305	Bd20070	pfam00464	SHMT	Serine hydroxymethyltransferase
Osi9812	Osj14203	Zm39491	2.42E + 08	Bd28258	pfam01406	tRNA-synt_1e	tRNA synthetases class I (C) catalytic
Osi9653	Osj44577	Zm33457	Sb5144	Bd6360	pfam00443	UCH	Ubiquitin carboxyl-terminal hydrolase
Osi2251	Osj35805	Zm98577	2.42E + 08	Bd20683	pfam12076	Wax2_C	WAX2 C-terminal domain
Osi15508	Osj14495	Zm95	Sb10474	Bd4536	pfam05495	zf-CHY	CHY zinc finger
Osi21052	Osj4519	Zm39479	Sb9831	Bd24331	No Pfam predicted		
Osi8778	Osj195	Zm100142	Sb1070	Bd2265	No Pfam predicted		
Osi20728	Osj16408	Zm34294	57806619	Bd19455	No Pfam predicted		
Osi17233	Osj20996	Zm100171	Sb1504	Bd16477	No Pfam predicted		
CT830510.1	Osj3010	Zm96019	Sb2210	Bd12504	No Pfam predicted		

### Conclusions

In the present work, we investigated the functional landscape of the four most important cereal plants *O. sativa* ssp *indica* and *japonica*, *S. bicolor* and *Z. mays* using the updated EST and mRNA sequences available in NCBI thus bridging the knowledge gap and updating the conserved AS catalog with functional elucidation. The availability of the conserved AS genes among the four cereal plants will facilitate to understand the regulation of the alternative physiological processes in global climate change biology and their subsequent impact on the genic-environmental interactions.

### Availability of supporting data

The data described in the work can be searched or downloaded at the Plant Alternative Splicing Database (http://proteomics.ysu.edu/altsplice/). Other detailed analysis data can be downloaded at http://proteomics.ysu.edu/publication/data/CerealAS/.

## Additional files

Additional file 1: Table S1. Protein family classification of alternative genes in cereal plants. (XLS 402 kb) Additional file 2: Table S3. Number of conserved alternative splicing genes in cereal crops. (XLS 31 kb) Additional file 3: Table S2. Conserved alternative splicing gene list in rice, sorghum, corn, and *Brachypodium distachyon*. (XLS 75 kb)

## Abbreviations

AltA: Alternative acceptor site
AltD: Alternative donor site
AS: Alternative splicing
CDS: Coding DNA sequence
ES: Exon skipping
IR: Intron retention
PUT: Putative unique transcript
ssp: Subspecies
